# Evaluation of the RAPID score as a predictor of postoperative morbidity and mortality in patients undergoing pulmonary decortication for stage III pleural empyema

**DOI:** 10.1016/j.clinsp.2024.100356

**Published:** 2024-04-11

**Authors:** Danilo Caribé Carneiro, Paula Duarte D'Ambrosio, Alessandro Wasum Mariani, Jaqueline Schaparini Fonini, Gabriela Ketherine Zurita Aguirre, João Pedro Carneiro Leão, Aurelino Fernandes Schmidt Júnior, Eihab O. Bedawi, Najib M. Rahman, Paulo Manuel Pêgo-Fernandes

**Affiliations:** a Thoracic Surgery Resident, Instituto do Coração, Hospital das Clínicas, Faculdade de Medicina, Universidade de São Paulo (HCFMUSP), São Paulo, SP, Brazil; b Thoracic Surgery Departament, Instituto do Coração, Hospital das Clínicas, Faculdade de Medicina, Universidade de São Paulo (HCFMUSP), São Paulo, SP, Brazil; c Department of Infection, Immunity and Cardiovascular Disease (IICD), University of Sheffield, Sheffield, United Kingdom of Great Britain and Northern Ireland United Kingdom; d Department of Respiratory Medicine, Sheffield Teaching Hospitals NHS Foundation Trust, Sheffield, United Kingdom of Great Britain and Northern Ireland United Kingdom; e Oxford Respiratory Trials Unit, Nuffield Department of Medicine, University of Oxford, NIHR Oxford Biomedical Research Centre, University of Oxford, Oxford, United Kingdom of Great Britain and Northern Ireland United Kingdom; f Oxford Centre for Respiratory Medicine, Oxford University Hospitals NHS Foundation Trust, Oxford, United Kingdom of Great Britain and Northern Ireland United Kingdom

**Keywords:** Empyema, Rapid score, Parapneumonic effusion, Surgery outcomes, Pleural disease

## Abstract

**Objective:**

: This study aims to correlate the RAPID score with the 3-month survival and surgical results of patients undergoing lung decortication with stage III pleural empyema.

**Methods:**

: This was a retrospective study with the population of patients with pleural empyema who underwent pulmonary decortication between January 2019 and June 2022. Data were collected from the institution's database, and patients were classified as low, medium, and high risk according to the RAPID score. The primary outcome was 3-month mortality. Secondary outcomes were the length of hospital stay, readmission rate, and the need for pleural re-intervention.

**Results:**

: Of the 34 patients with pleural empyema, according to the RAPID score, patients were stratified into low risk (23.5 %), medium risk (47.1 %), and high risk (29.4 %). The high-risk group had a 3-month mortality of 40 %, while the moderate-risk group had a 6.25 % and the low-risk group had no deaths within 90 days, confirming a good correlation with the RAPID score (*p* < 0.05). Sensitivity and specificity for the primary outcome in the high-risk score were 80.0 % and 79.3 %, respectively. The secondary outcomes did not reach statistical significance.

**Conclusions:**

: In this retrospective series, the RAPID score had a good correlation with 3-month mortality in patients undergoing lung decortication. The morbidity indicators did not reach statistical significance. The present data justifies further studies to explore the capacity of the RAPID score to be used as a selection tool for treatment modality in patients with stage III pleural empyema.

## Highlights

• This study establishes a strong correlation between the RAPID score and 3-month mortality in patients undergoing lung decortication for pleural empyema.
• Patients were stratified into low, medium, and high-risk groups based on the RAPID score, demonstrating that this approach can be valuable in identifying patients with a higher likelihood of complications. This can inform treatment planning and post-operative monitoring.
• While the results suggest a strong correlation, prospective studies are needed to fully validate the use of the RAPID score in this population. This underscores the importance of future clinical research to enhance the selection of the initial treatment for patients with pleural empyema.

## Introduction

Pleural empyema is a prevalent and frequently life-threatening condition, accounting for 1 million hospitalizations annually in Europe (1)^.^ This ailment is associated with substantial mortality rates reaching up to 20 % (2). The fundamental approach to managing pleural infections involves appropriate antibiotic therapy, clinical support, and timely drainage (2, 3, 4, 5, 6, 7, 8, 9). Surgery, serving as a rescue therapy in approximately 30 % of cases, becomes particularly relevant for patients in stages 2 or 3, aiming to reduce hospitalization and enhance clinical outcomes (10,11).

However, the optimal strategy for stage III empyema for pulmonary decortication remains complex (12), especially in severely ill patients who cannot tolerate the decortication, the ideal procedure, due to its aggressive nature. The alternatives to pulmonary decortication are mainly prolonged tube drainage or open thoracostomy, both associated with a significant impact on quality of life.

Nevertheless, the indiscriminate application of surgical intervention in all pleural infection cases is not justifiable, given its association with significant morbidity, including perioperative and anesthetic mortality (13, 14, 15). The criteria for selecting patients who would benefit most from surgery due to an increased likelihood of clinical treatment failure remain unanswered. Consequently, the appropriateness of surgical intervention based on the patient's clinical condition lacks a precise definition, and clinical practices vary according to individual surgical preferences.

To date, the RAPID score (Renal, Age, fluid Purulence, Infection source, Dietary [albumin]) is a validated scoring system that allows risk stratification in patients with pleural infection at presentation (16). The score categorizes patients into low (0–2), medium (3–4), and high (5–7) risk groups. A higher score is linked to elevated 3- and 12-month mortality rates and prolonged hospitalization (16). Despite promising results in studies assessing the RAPID score, its adoption in clinical practice remains infrequent, with no validation in diverse populations, such as the Brazilian population. Moreover, the authors saw the potential of the RAPID score to be used for surgeons to decide between surgical decortication or alternative procedures in patients with stage III pleural empyema. But first, due to the lack of literature, it is necessary to demonstrate the quality of the RAPID score as a risk predictor tool in this specific population.

Therefore, the objective of this study is to assess the RAPID score as a predictor of morbidity and mortality in a retrospective cohort of patients with pleural empyema undergoing pulmonary decortication in a Brazilian population.

## Materials and methods

**Fig. 1:**
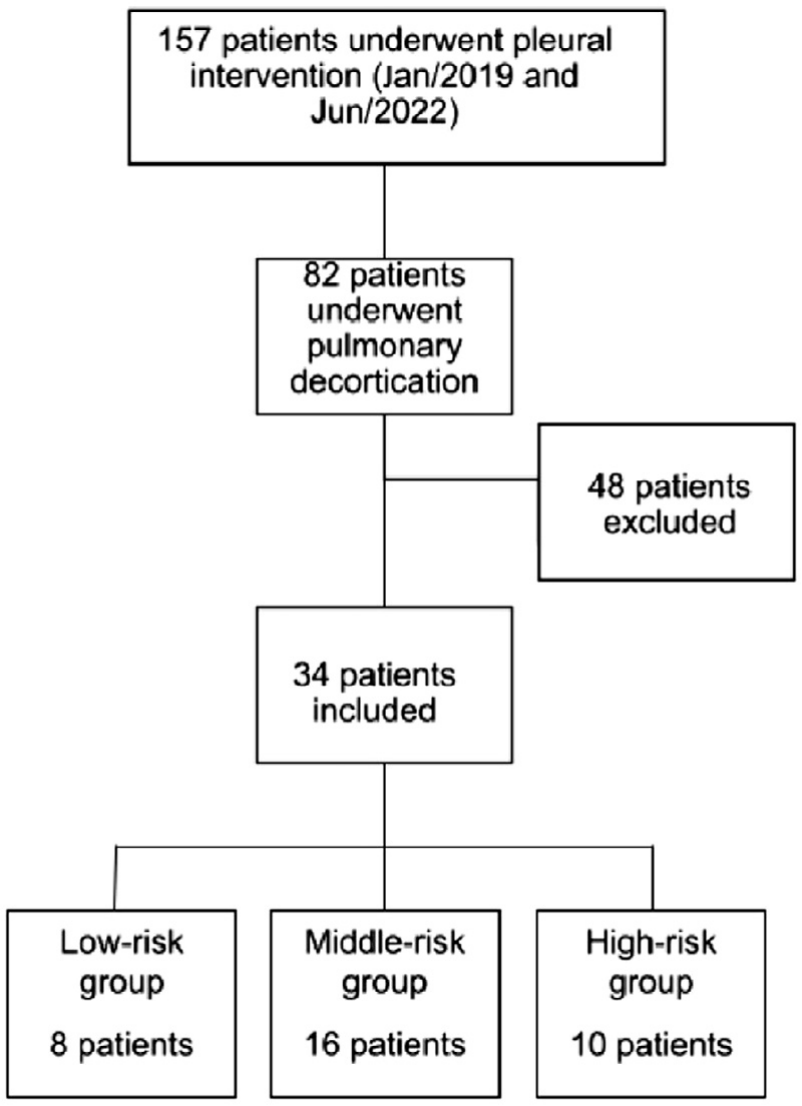
Study selection flowchart.

This retrospective study was performed at a Quaternary Teaching Hospital in São Paulo, Brazil. The Institutional Review Board approved the study (CAPPesq approval 33,365,720.2.0000.0068). The authors retrospectively reviewed the electronic medical records for the thoracic surgery database for those who had undergone pulmonary decortication for primary empyema between January 2019 and June 2022. The study population consisted of all patients receiving decortication for empyema secondary to pneumonia. Inclusion criteria: Patients who were submitted to pleural drainage before the surgical procedure and presented with purulent fluid or positive culture test were included. Patients with a clinical picture highly suggestive of pleural empyema but who had not undergone any pleural fluid analysis or whose pleural fluid analysis had complicated pleural fluid, according to Light criteria (8), were also included. Patients younger than 18 years of age or with previous pulmonary resection, less than 3 months life expectancy, history of primary pulmonary neoplasia, non-parapneumonic etiology, or incomplete data were excluded from the study (Fig. 1). Board-certified thoracic surgeons performed all thoracic surgical procedures.

### Data collection

Basic demographic information, including age, sex, comorbidities, smoke history or alcoholism, and SARS-CoV-2 infection were extracted from electronic medical records. Pleural drainage, when indicated, was done by the Thoracic Surgery Service, and the anatomical location of placement and the chest tube type and diameter varied according to each patient's necessity. Pre-operatory data were antibiotic therapy and length of stay with pleural drain. Charlson score and RAPID score were calculated for all patients.

Variables collected include surgical technique, conversion rate, number of chest tubes, chest tube duration, length of hospital and ICU stay, reintervention, hospital readmission in 30 days and 3-month survival.

### Surgical procedure

The procedure was performed in a standard operating room under sterile conditions. Patients underwent general anesthesia with a double-lumen endotracheal tube allowing for selective single-lung ventilation. Surgery was performed in the lateral decubitus position. The surgical procedure was performed by thoracotomy or Videothoracoscopy (VATS), decided by the surgeon. Evacuation of the fluid components of the empyema was the first step, followed by decortication of the lung and dissecting the empyema's capsule of the parietal, mediastinal, and diaphragmatic pleura.

One or two chest tubes were placed at the end of the operation. Negative pressure was not routinely used. Chest tubes were removed gradually after air leakage stopped, the fluid collection was below 100 mL per day and the X-Ray demonstrated adequate lung expansion.

### RAPID score

The RAPID (Renal (urea), Age, fluid Purulence, Infection source, and Dietary (albumin) score at baseline presentation was calculated and from the derived score, patients were placed in one of three risk categories (low, medium, or high) for analysis, according to the original paper (16). Individual patients did not have the RAPID score calculated or used to guide their clinical management during the study.

The score was created using the two largest multicenter studies of pleural infection (MIST-1 and MIST-2)(17,18) to create clinically accessible predictors (Table 1) associated with 3-month mortality.

**Table 1:** The RAPID Score parameters.

Parameter	Score
Renal (urea) mg/dL	
< 14	0
14‒23	1
> 23	2
Age(years)	
< 50	0
50‒70	1
> 70	2
Purulence of pleural fluid	
Purulent	0
Non-purulent	1
Infection setting	
Community-acquired	0
Hospital = Acquired	1
Dietary factors (serum albumin) mg/dL	
≥ 2.7	0
< 2.7	1

The risk model developed gave more weight to both age and urea because of their high odds ratios for mortality, with the other three variables scoring the same. Therefore, each patient's RAPID score ranged between 0 and 7, with low-risk patients (score 0‒2) having a 1 % to 3 % mortality at 3 months compared to 31 % to 51 % for high-risk patients. risk (score 5‒7) (16).

The primary outcome was 3-month mortality. Secondary outcomes were the length of hospital stay, readmission rate, and the need for additional intervention.

### Statistical analysis

Descriptive statistical analysis was used to summarize the characteristics of the studied patients and surgical procedures. Frequencies and percentages are presented for categorical variables, and continuous variables are summarized as the median. Multiple variable models used logistic regression. Variables with *p* < 0.05 in univariable analysis were retained in the final model. All statistical analyses were performed using the software SPSS (IBM Corp) version 20.0. Probability (p) values of less than 0.05 were considered statistically significant.

## Results

Thirty-four patients were included in the study, of which 26 were men (76 %). The mean age was 49.7 years (±16.0). Twenty-one patients (61.8 %) had associated comorbidities, diabetes mellitus in 16 patients (47.1 %) and coronavirus infection in eight patients (23.5 %). Twenty-one (61.8 %) patients underwent pleural drainage before surgical intervention. The surgical procedure was performed by VATS in 31 patients (91.2 %), with one conversion to thoracotomy (Table 2).

**Table 2:** Baseline characteristics of study participants.

Demographic characteristics	
Age (years)	50 (±16.1)
Male sex	26 (76.5%)
Source of infection	
Community-acquired	14 (42.4 %)
Hospital-acquired	19 (57.6 %)
Smoking history	6 (17.6 %)
Etilism	3 (8.8 %)
SARS-CoV-2 infection	8 (23.5 %)
Previous pleural drainage	21 (61.8 %)
Laboratorial analyses	
WBC (cel/mm^3^)	14.132 ± 5.758
Reactive C-protein (mg/L)	175.2 ± 144.0
Serum albumin (mg/dL)	2.5 ± 0.6
Pleural LDH (units/L)	3.796 ± 3.401
Plural glicose	45.3 ± 62.0
Comorbidities	
Endocrine	16 (47.1 %)
Cardiac	11 (32.4 %)
Pulmonary	6 (17.6 %)
Renal	1 (2.9 %)

According to the RAPID score, 8 patients (23.5 %) were stratified as low-risk, 16 as medium-risk (47.1 %), and 10 as high-risk (29.4 %). The mean length of hospital stay for the high-risk group was 54.6 days (±30.0), while for the medium and low risk, was 42 days for both (*p* = 0.283).

The Charlson score classified all patients. In the low-risk group, the average score found was 0.5 points, while the medium and high-risk groups were 1.43 points and 3.4 points, respectively. Evidencing association between the RAPID score and the Charlson score.

**Fig. 2:**
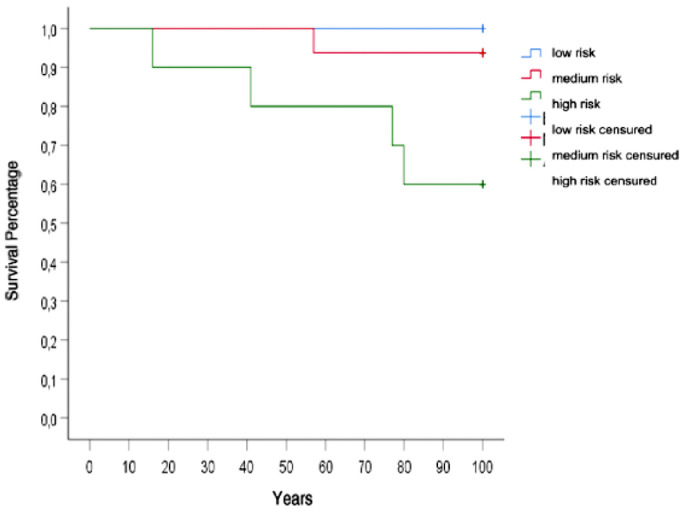
Kaplan-Meier survival among RAPID-score risk groups.

When the univariate analysis was performed, the presence of single or multiple pleural collections on chest CT (*p* = 1.00), as well as the presence of pleural thickening (*p* = 0.559), was not related to patient survival at 90 days nether with the RAPID score classification. There were 3 readmissions in the 30-day period, two in the high-risk group and one case in the moderate-risk group. Additional procedures were required in 6 patients (17.6 %). Although RAPID score was correlated with surgical reintervention, length of stay, and readmission within 30 days, statistical significance was not reached for these outcomes (*p* = 0.513) (Table 3). The high-risk group had a 3-month mortality of 40 %, while the moderate-risk group had 6.25 %, and low risk had no deaths within 90 days, showing a good correlation between the RAPID score and 3-month survival (*p* < 0.05) (Table 4, Fig. 2).

**Table 3:** Secondary outcomes according to baseline RAPID risk category.

	RAPID score			
Variable n (%)	Low risk	Medium risk	High-risk	Total
Reintervention	1 (12,5)	2 (12.5)	3 (30.0)	6 (17.6) (*p* = 0.513)
Readmissions in (30-day period)	0 (0.0)	1 (6.3)	2 (20.0)	3 (8.8) (*p* = 0.425)
	Mean (S.D)			
Hospital stay	42.0 (±15.4)	42.0 (±31.0)	54.6 (±30.0)	*p* = 0.283

**Table 4:** Association between RAPID score and 3-month mortality.

	RAPID score			
Survival n (%)	Low risk	Medium risk	High risk	Total
≤ 3-month	0 (0)	1 (6.3)	4 (40)	5 (14.7)
> 3-month	8 (100.0)	15 (93.8)	6 (60)	29 (85.3)
Total	8 (100)	16 (100)	10 (100)	34 (100)
	Fisher Exact Test = 5.681			^a^*p* = 0.047

^a^p Fisher exact test significance probability.

In the multivariate analysis, only two variables showed a statistically significant association with 3-month mortality. The presence of normal white blood cell values (RV 10,500 cel/mm^3^) was associated with increased 3-month mortality (*p* = 0.049), with an OR of 18.9 (95 % IC 1.01, 353.9). Comparing the low and medium-risk groups, the high-risk group presented as an essential risk factor for mortality in the 90-day period, with an odds ratio of 30.1 (95 % IC 1.7, 545.3) (*p* = 0.021). Sensitivity and specificity for the primary endpoint at high-risk score were 80.0 % and 79,3 %, respectively.

## Discussion

In 2014, Rahman et al. (16) developed a prognostic model to predict 3-month mortality in patients with pleural infections at their diagnosis. The model, known as RAPID Score, was derived using data from the MIST1 clinical trial (7) and validated on the MIST2 cohort and the PILOT cohort (17,19). The scoring system accurately prognosticates short-term mortality but did not specifically address surgically treated empyema patients. In this sense, this study is the first to retrospectively evaluate the performance characteristics of the RAPID scoring system in a cohort of 34 patients with pleural infections who underwent pulmonary decortication.

Evaluating the severity of patients in the present study, all were classified according to the Charlson score. The average score was 1.8 points, of which 29.4 % of the patients did not score, 35.2 % had 1 or 2 points, and 35.2 % had 3 points or more. On the other hand, Nayak and colleagues, in a retrospective cohort of 9.014 patients diagnosed with empyema between 1995 and 2015, presented a Charlson Comorbidity Index with more than 3 points in less than 25 % of patients in all analyzed groups (20), suggesting that the patients in this study had a non-negligible severity.

The exact inference can be made regarding the analysis of mortality. In a retrospective study done by Semenkovich et al. between 2009 and 2014, which evaluated the treatment of 4.095 patients diagnosed with empyema, the mortality rate observed in the VATS group was 6.3 %, and the open access group was 7.5 % (21), significantly lower than that found in the present study, which was 14.7 %.

In a recent analysis of the Society of Thoracic Surgeons database, 7312 patients who underwent pulmonary decortication due to parapneumonic empyema were retrospectively referred. In this study, Tower et al., based on the multivariate analysis of their data, identified that several factors are associated with poor outcomes after pulmonary decortication. The main factors associated with a higher rate of complications and mortality were age, comorbidities (mainly severe renal dysfunction or need for preoperative dialysis), and poor functional status (22).

The RAPID score objectively covers these factors found by Tower et al. In the multivariate analysis, the authors observed a significantly increased risk of postoperative mortality in patients classified as high-risk compared with low and medium-risk groups (OR = 30.1). However, in this series, no association was observed between the RAPID score and the re-approach rate, 30-day readmission, length of hospital stay, or length of ICU stay.

Due to its retrospective nature and the small sample, this study cannot fully validate the applicability of the RAPID score as a tool to predict mortality risk at 3 months in patients undergoing pulmonary decortication with parapneumonic empyema. There is a need to carry out more extensive studies to validate the RAPID score in this population and, furthermore, the possibility to tailor the surgical treatment choice in adequacy to the mortality risk. From this point of view, the RAPID score can become an essential tool for defining surgical indications in the face of parapneumonic empyema, mainly in the high-risk group.

## Conclusion

The RAPID score had an excellent correlation with 3-month mortality for surgical patients in the Brazilian population. The morbidity indicators, namely length of hospital stay, readmission rate, and the need for pleural re-intervention, did not reach statistical significance. The present data is the first to highlight the usefulness of the RAPID score for surgical patients and justifies further studies to explore the capacity of the RAPID score to be used as a selection tool for treatment modality in patients with stage III pleural empyema.

## CRediT authorship contribution statement

**Danilo Caribé Carneiro:** Methodology, Validation, Formal analysis, Writing – review & editing, Visualization. **Paula Duarte D'Ambrosio:** Validation, Formal analysis, Writing – review & editing, Visualization. **Alessandro Wasum Mariani:** Supervision, Project administration. **Jaqueline Schaparini Fonini:** Investigation, Writing – original draft, Writing – review & editing. **Gabriela Ketherine Zurita Aguirre:** Conceptualization, Methodology. **João Pedro Carneiro Leão:** Conceptualization, Methodology. **Aurelino Fernandes Schmidt Júnior:** Writing – review & editing. **Eihab O. Bedawi:** Writing – review & editing. **Najib M. Rahman:** Writing – review & editing. **Paulo Manuel Pêgo-Fernandes:** Supervision.

## Declaration of competing interests

The authors declare no conflicts of interest.
